# A patient-reported outcome measure for patients with pituitary adenoma undergoing transsphenoidal surgery

**DOI:** 10.1007/s11102-022-01251-x

**Published:** 2022-07-15

**Authors:** Elika Karvandi, John Gerrard Hanrahan, Danyal Zaman Khan, Pierre-Marc Boloux, Fion Bremner, Ivan Cabrilo, Neil Dorward, Joan Grieve, Sue Jackson, Glenda Jimenez, Inma Serrano, Victoria Anne Nowak, Angelos Kolias, Stephanie E. Baldeweg, Hani Joseph Marcus

**Affiliations:** 1 Department of Neurosurgery, University of Cambridge, Cambridge, UK; 2 Division of Neurosurgery, National Hospital for Neurology and Neurosurgery, London, UK; 3 Wellcome/EPSRC Centre for Interventional and Surgical Sciences, University College London, London, UK; 4 Department of Diabetes & Endocrinology, University College London Hospitals NHS Foundation Trust, London, UK; 5 Department of Neuro-Ophthalmology, National Hospital for Neurology and Neurosurgery, London, UK; 6 Department of Psychology, University of Plymouth, Plymouth, UK; 7 Centre for Obesity and Metabolism, Department of Experimental and Translational Medicine, Division of Medicine, University College London, London, UK

**Keywords:** Pituitary adenoma, Patient-reported outcome measure, PROM, Transsphenoidal surgery, Quality of life, Pituitary surgery

## Abstract

**Purpose:**

Pituitary adenomas affect patients’ quality-of-life (QoL) across several domains, with long-term implications even following gross-total resection or disease remission. While clinical outcomes can assess treatment efficacy, they do not capture variations in QoL. We present the development and validation of a patient reported outcome measure (PROM) for patients with pituitary adenomas undergoing transsphenoidal surgery.

**Methods:**

The COSMIN checklist informed the development of the pituitary outcome score (POS). Consecutive patients undergoing surgical treatment for suspected pituitary adenoma at a single centre were included prospectively. An expert focus group and patient interviews informed item generation. Item reduction was conducted through exploratory factor analysis and expert consensus, followed by assessment of the tool’s validity, reliability, responsiveness, and interpretability.

**Results:**

96 patients with a median age of 50 years validated the POS. The final questionnaire included 25 questions with four subscales: EQ-5D-5L-QoL, Visual Symptoms, Endocrine Symptoms and Nasal Symptoms.

**Conclusion:**

The POS is the first validated PROM for patients undergoing transsphenoidal surgery for a pituitary adenoma. This PROM could be integrated into contemporary practice to provide patient-centred outcomes assessment for this patient group, aligning more closely with patient objectives.

**Supplementary Information:**

The online version contains supplementary material available at 10.1007/s11102-022-01251-x.

## Introduction

Pituitary adenomas comprise 18% of central nervous system tumours [1], with a prevalence of 75.7–115.6/100,000 [2]. Surgical resection is the mainstay of treatment for symptomatic non-functioning and certain functioning adenomas. Transsphenoidal surgery is the preferred surgical approach, with relatively lower complication rates, shorter operative times and faster recovery compared with alternatives [3–5].

Surgical outcome measures of treatment effectiveness include the extent of surgical resection, biochemical remission, visual recovery, and re-operation rates [6–9]. Yet, there is growing recognition of the wider impact of pituitary adenomas on patient quality of life (QoL) across multiple domains [10–12]. Their long-term impact adversely affects patients’ QoL despite gross-total resection or induction of remission [13, 14], meaning established signifiers of “effective” treatment fail to capture variations in patients’ experience or long-term QoL. Whilst achieving existing clinical outcome measures is fundamental, particularly in disease causing significant morbidity, alone they are insufficient to assess quality of care [15].

Patient-reported outcome measures (PROMs) are established markers of treatment efficacy [16, 17]. Both generic and disease-specific PROMs have been used to evaluate pituitary adenoma surgery outcomes [18, 19]; however, existing efforts are limited to disease specific tools [18] or employment of employ generic patient-reported outcome measures [20]. The Leiden Bother and Needs questionnaire (LBNQ-Pituitary) assesses how much patients are affected by their condition [21], but provides limited assessment on visual symptoms and lacks key items related to nasal symptoms, associated with non-functioning adenomas and transsphenoidal surgery, respectively.

Currently there is no gold-standard, dedicated PROM for patients undergoing surgery for pituitary adenoma. There is value in developing such a PROM, considering the impact pituitary adenomas have on QoL. Establishing a validated PROM would greatly facilitate outcomes research and align interventions with patient priorities. Individualised patient feedback can drive improvements in clinical care and inform providers regarding performance metrics [22].

We aimed to produce a validated PROM tool for patients with pituitary adenoma undergoing surgery following the COnsensus-based Standards for the selection of health Measurement INstruments (COSMIN) checklist [23, 24].

## Methods

### Study design

The PROM questionnaire, named the Pituitary Outcome Score (POS), was developed and validated according to the COSMIN checklist [25] using four steps (1) identification of patient population, (2) item generation, (3) item reduction and (4) determination of its validity, reliability, responsiveness, and interpretability.

### Patient identification and participation

Prospective, consecutive, eligible patients with pituitary adenomas attending an elective neurosurgical clinic and scheduled for transsphenoidal surgery between February 2019 and August 2021 were invited to participate in the study. Verbal consent was obtained, and surveys were distributed. Inclusion criteria were: minimum age 18, diagnosed with pituitary adenomas, scheduled for transsphenoidal surgery, and able to complete the questionnaire.

### Item generation

Prior to developing the POS questionnaire, a systematic review of the literature was conducted to identify existing PROMs used for the target patient population. An expert focus group developed the items relevant for the questionnaire, followed by patient interviews. We incorporated established QoL questions into the POS from the EQ-5D-5L[26], here referred to as the EQ-5D-5L-QoL.

#### Expert focus group

The expert focus group comprised three endocrinologists, three neurosurgeons, two ophthalmologists, one psychologist and one specialist nurse. All participating members have extensive experience in managing patients with pituitary adenomas undergoing transsphenoidal surgery. Following discussion, items were developed by four members of the group and reviewed by all group members.

#### Patient interviews

Involvement of patients during the development of PROMs ensures an accurate reflection of patient experience [24, 27]. In the final stage of Item Generation, patients who had previously undergone transsphenoidal surgery for pituitary adenomas (invited from The Pituitary Foundation) were interviewed to review the questionnaire’s readability, and ease of comprehension.

#### Data collection

Participants completed the surveys at four different time points; (1) pre-operatively: the 36-item Short Form (SF-36) [28], a Global Perceived Effect (GPE) scale, demographics, and the preliminary POS, followed by (2) two weeks later: the preliminary POS and a GPE scale, as well as a (3) 3-month post-operation POS and GPE scale, and a (4) 6-month post-operative POS and a GPE scale. Surveys were completed by participants in their own time.

The GPE scale, a single-item global rating scale, quantifies the patient’s perception of their current overall health and provides a measure of perceived recovery [29, 30]. The GPE was adjusted for each survey time-point to reflect the stage of their condition. The rating scale included in the first survey assessed the patient’s perception of the degree to which the pituitary adenoma affected their overall health. The GPE scales in the three other surveys assessed current health status compared with the first survey timepoint (Online Resource 1). In addition to providing a measure for patient perceived recovery, the GPE was included to serve as an anchor for the responsiveness and interpretability analyses.

### Item reduction and measurement properties

A prospective clinical cohort study was conducted. The measurement properties (reliability, responsiveness, validity, and interpretability) of the POS were assessed according to the COSMIN checklist. Internal consistency, test–retest reliability, and measurement error were calculated to assess the reliability of the questionnaire, while the validity was evaluated using calculated correlation coefficients with a generic PROM, the SF-36. Responsiveness was assessed by calculating the mean change scores (MCS) for patients reporting improved, no different and deteriorated health-states on the GPE six months after surgery, as well as the standardised response mean (SRM) and effect size. Furthermore, assessment of interpretability was done through examining minimal important difference (MID), minimal important change (MIC), smallest detectable change (SDC), and floor and ceiling effects. Statistical analyses were performed for each subscale using SPSS statistics (version 27).

#### Item reduction

An exploratory factor analysis (EFA) [31] was done to assess the structural validity of the questionnaire using principal axis factoring with promax rotation. Following data collection completion, item reduction was discussed in the expert focus group and consensus regarding item removal was achieved, guided by EFA results and clinical judgement.

#### Reliability

Cronbach’s alpha was calculated using the baseline POS data to evaluate internal consistency, where scores below 0.50 and above 0.70 were considered to indicate poor and acceptable internal consistency, respectively [32]. Test–retest reliability was evaluated by calculating the intraclass correlation coefficient (ICC) using the two pre-operative surveys, baseline and the retest two weeks after. Only participants reporting no change in their clinical condition on the GPE scale were included for the test–retest reliability assessment. The two-week time interval was chosen as it was deemed long enough for patients to have forgotten their previous responses, while being short enough for their condition to have remained stable. The ICC two-way mixed effect with absolute agreement was calculated with a score above 0.70 indicating a good test–retest reliability [33]. The degree of variation in the measurement error of the POS subscales was determined with standard error of measurement (SEM) and was calculated using the formula SEM = SD*(√(1-ICC)).

#### Validity

The POS was correlated with the SF-36 questionnaire, a generic PROM measuring health status consisting of eight subscales. All items carry equal weight and are scored on a scale of 0–100. The SF-36 is commonly used as it is a valid and reliable measurement for health-related QoL [34].

The Spearman’s correlation coefficients were calculated for individual POS subscales with individual SF-36 subscales from the baseline survey. The correlation coefficients were used to determine the degree of relation between subscale of the two questionnaires, where constructs are either related (convergence) or not related (divergence), and whether this aligned with theoretical expectations [35]. Correlations 0 to  ± 0.40 were considered low, correlations between ± 0.40 and  ± 0.70 were considered moderate, and correlations ± 0.70 to  ± 1 were considered high [36].

We expected related constructs would be more strongly correlated than unrelated constructs. The a priori hypothesis was that a stronger correlation would be observed between the POS EQ-5D-5L-QoL subscale and the SF-36 overall, while the Endocrine Symptoms subscale would be moderately correlated with the SF-36 subscales. Furthermore, we expected a weaker correlation would be observed with the Visual Symptoms and Nasal Symptoms subscales as these measure pituitary adenoma-specific and surgery-specific properties.

#### Responsiveness

The post-operation GPE score, where patients rate their condition on a scale ranging from “much better” to “much worse”, aided evaluation of the questionnaire’s responsiveness. The Spearman’s correlation coefficient was calculated from the baseline questionnaire and the 6-month post-operative questionnaires. The degree of change was measured using the participants’ responses to the GPE scale and grouped as improved, no change, and deteriorated. The a priori hypothesis was that the change scores between the baseline and the post-operative POS questionnaires would correlate with the GPE responses. Furthermore, the SRM and effect size were calculated according to each GPE anchor, where an effect size of > 0.80 is generally considered a large change [34, 37].

#### Interpretability

Interpretability was evaluated with the SDC, MIC, MID, and floor and ceiling effects. The SDC was calculated to determine the smallest change in scores needed to identify whether the change in questionnaire score is real beyond the measurement error. The SDC was calculated at the individual level (SDC(ind)) and the group level (SDC(gr)) using the SEM of the test–retest reliability. The SDC was calculated at the individual level as SEM*1.96*√2, and calculated at the group level as (SEM*1.96*√2)/n. The MIC and MID were calculated using anchor-based methods where the GPE score was the external criterion. The MIC was reported as the mean POS change score for patients answering “better” on the GPE scale. The MID was calculated by subtracting the mean POS change score for patients reporting “no change” on the GPE scale from the MIC. To distinguish with certainty between clinically important changes and measurement errors, the MIC needs to be bigger than the SDC (ind) [38]. A floor and ceiling effect was considered to have occurred if more than 15% of participants reported the lowest or highest possible score for a subscale.

### Other analyses

Mean subscale scores were calculated for baseline and 6-month follow-up surveys. Two sample t-tests were done to compare the mean POS subscale scores between patients who underwent an endoscopic approach and those that underwent a microscopic approach. A p value ≤ 0.05 was considered to be significant.

## Results

### Patient identification and participation

96 patients were recruited (median age 50 years, slight female predominance 53.1%). There were 76 macroadenomas (81.7%) and 17 microadenomas (18.3%). Histology for three patients was unexpected: meningioma (n = 1) or Rathke’s Cleft cyst (n = 2). 54 patients had non-functioning and 42 had functioning adenomas. 90 patients underwent surgery, with 46 undergoing endoscopic resection and 44 undergoing microscopic resection (Table 1).

**Table 1:** Demographics

Characteristic	N (%)
*Demographics*	
Sex: female, N (%)	51 (53.1%)
Median age in years (IQR)	50 (18.3)
*Tumour size* (Adenoma, N = 93)	
Micro (0–10 mm) Macro (> 10 mm)	17 76
*Diagnosis*	
Functioning adenoma Acromegaly Cushing’s disease Prolactinoma Mixed (growth hormone-secreting/prolactinoma) Thyrotropinoma	42 16 15 5 1 1
Non-functioning adenoma	54
Meningioma	1
Rathke’s cleft cyst	2
*Surgery*	
Underwent surgery Endoscopic Microscopic	90 46 44

Following removal of entries with incomplete survey completion, the baseline survey had 92 participants (95.8%), the retest survey had 60 participants (89.6%), the 3-month follow-up survey had 57 participants (91.9%), and the 6-month follow-up survey had 44 participants (95.7%). There were no missing data entries for participants included in the analyses. However, data numbers had to be adjusted for eligibility when performing the analyses resulting in lower sample sizes.

### Item generation

#### Expert focus group

The first version of the questionnaire comprised 15 items with four subscales: EQ-5D-5L-QoL, Visual Symptoms, Endocrine Symptoms, and Nasal Symptoms. The EQ-5D-5L [39] was integrated in the POS as a QoL measure. Three items from the Cat-PROM5 were rephrased and included in the Visual Symptoms subscale [40]. Following review to assess for readability and ease of comprehension by the focus group, nine additional items were added.

#### Patient interview

Data saturation was achieved after four patient interviews. Two questions were added, use of capital letters was minimised and sentences were shortened. A clarifying sentence stating patients should select the option “no problem at all” for questions they considered to be unrelated to them was added.

#### Preliminary questionnaire pilot

The fourth version of the questionnaire contained 26 questions with four subscales: EQ-5D-5L-QoL (five items), Visual Symptoms (four items), Endocrine Symptoms (14 items) and Nasal Symptoms (three items). The questionnaire was piloted with two additional patients; no further modifications were needed, so the fourth version of the questionnaire was used in this study.

### Item reduction and measurement properties

#### Item reduction

The fourth version of the POS comprised 26 items. Following review of the EFA results and clinical judgement, one item (“how much are you having problems recovering from minor illnesses”) was removed from the Endocrine subscale. 25 items were included in the final version of the POS (panel) and subsequent analyses were performed on these data.

Panel: Pituitary Outcome Score.

#### Reliability

Cronbach’s alpha was above 0.70 for the EQ-5D-5L-QoL, Visual and Endocrine subscales indicating good internal consistency [32]. The Nasal subscale had a Cronbach’s alpha of 0.63 which is acceptable [41].

Participants who completed both the baseline and retest survey, as well as stating “same as before” on the GPE scale, were included in the test–retest analysis, yielding a sample size of 37. The ICC values were above 0.90 for all subscales (Table 2) indicating excellent reliability [33]. The standard error of measurement is given in Table 2.

**Table 2:** Reliability

POS Subscale	Cronbach’s alpha, n = 92	ICC (95% CI), n = 37	SEM
EQ-5D-5L-QoL	0.835	0.956 (0.910–0.978)	1.49
Visual symptoms	0.759	0.912 (0.829–0.954)	1.66
Endocrine symptoms	0.910	0.954 (0.911–0.976)	5.81
nasal symptoms	0.634	0.908 (0.821–0.953)	1.76

*EQ-5D-5L-QoL* EuroQol-5 dimention-5 level-quality of life, *ICC* Intraclass correlation coefficient, *POS* Pituitary outcome score, *SEM* Standard error of measurement

#### Validity

The POS EQ-5D-5L-QoL subscale had correlation coefficients below − 0.60 with every SF-36 subscale of which three were below − 0.70, while the POS Endocrine subscale had correlation coefficients below − 0.50 with every SF-36 subscale. As expected, the correlation coefficients between the Visual Symptoms subscale and SF-36 subscales were all above − 0.40. The Nasal subscale had low to moderate correlations with the SF-36 with five subscales having coefficients above − 0.40 (Table 3).

**Table 3:** Construct validity

	POS subscales, n = 92			
	EQ-5D-5L-QoL	Visual symptoms	Endocrine symptoms	Nasal symptoms
SF36				
General health	− 0.686**	− 0.095	− 0.627**	− 0.280**
Physical function	− 0.767**	− 0.264*	− 0.560**	− 0.356**
Physical role	− 0.759**	− 0.242*	− 0.644**	− 0.420**
Emotional role	− 0.614**	− 0.109	− 0.681**	− 0.385**
Social functioning	− 0.689**	− 0.214*	− 0.745**	− 0.451**
Bodily pain	− 0.738**	− 0.319**	− 0.655**	− 0.609**
Vitality	− 0.692**	− 0.002	− 0.728**	− 0.344**
Mental health	− 0.600**	− 0.141	− 0.735**	− 0.357**

*EQ-5D-5L-QoL* EuroQol-5 dimention-5 level-quality of life, *POS* Pituitary outcome score, *SF36* 36-Item short form

*Correlation is significant at the 0.05 level (2-tailed)

**Correlation is significant at the 0.01 level (2-tailed)

#### Responsiveness

The change in scores from baseline to the 6-month post-operation survey (Table 4) showed low correlation with GPE scores. Overall, the MCS were higher for patients reporting feeling ‘better’ and ‘much better’ on the GPE scale post-operation, meaning they had a greater reduction in their POS subscale scores, except for the Nasal subscale where the ‘no change’ group had a higher MCS. Effect size was above 0.80 for the EQ-5D-5L-QoL, Visual and Endocrine subscales for the improved group and the Endocrine subscale for the deteriorated group.

**Table 4:** Responsiveness

POS Subscales	Spearman’s, n = 38	Improved group^§^, n = 29 (76%)			No change group^§§^, n = 5 (13.2%)			Deteriorated group^§§§^, n = 4 (10.5%)		
		MCS	Effect size	SRM	MCS	Effect size	SRM	MCS	Effect size	SRM
EQ-5D-5L-QoL	− 0.356*	2.48	1.04	0.94	1.60	0.35	0.47	1.00	0.22	0.43
Visual symptoms	− 0.198	1.86	0.88	0..85	1.40	0.33	0.37	− 1.00	− 0.33	− 0.23
Endocrine symptoms	− 0.320	10.21	1.07	1.16	3.80	0.30	0.79	6.25	0.92	0.73
Nasal symptoms	− 0.221	0.52	0.22	0.19	1.00	0.30	0.42	0.00	0	0

*EQ-5D-5L-QoL* EuroQol-5 dimention-5 level-quality of life, *MCS* Mean change score, *POS* Pituitary outcome score, *SRM* Standardised response mean

*Correlation is significant at the 0.05 level (2-tailed)

^§^GPE response = “much better” or “better”

^§§^GPE response = “same as before”

^§§§^GPE response = “much worse” or “worse”

#### Interpretability

The values for the MIC and MID for the 6-months post-operation timepoint and values for the individual and group SDCs are given in Table 5.

**Table 5:** Interpretability

	6-months				Baseline survey, n = 92		6-month post-operation, n = 44	
POS subscales	MIC, n = 16	MID, n = 5	SDC(ind), n = 37	SDC(gr), n = 37	Floor effect %	Ceiling effect %	Floor effect %	Ceiling effect %
EQ-5D-5L-QoL	2.00	0.40	4.13745561	0.68019351	9	0	25	0
Visual symptoms	1.71	0.21	4.58906731	0.75443802	9	0	18	0
Endocrine symptoms	7.43	3.63	17.2124842	2.82971498	0	1	0	0
Nasal symptoms	0.14	− 0.86	4.88809919	0.80359856	20	1	23	0

*EQ-5D-5L-QoL* EuroQol-5 dimention-5 level-quality of life, *MIC* Minimal important change, *MID* Minimal important difference, *POS* Pituitary outcome score, *SDC(gr)* Smallest detectable change, group level, *SDC(ind)* Smallest detectable change, individual level

No subscale had a ceiling effect for any survey (Table 5). The Nasal subscale had a floor effect on both surveys while the EQ-5D-5L-QOL and Visual subscales had floor effects on the post-operation survey.

### Other analyses

There were no significant differences in POS subscale scores between the endoscopic approach and microscopic approach (Table 6).

**Table 6:** Other analyses

POS Subscale	Mean baseline survey score	Mean 6-month post-op survey score	Endoscopic (n = 20) vs microscopic (n = 21) surgery scores P value (95% CI)
EQ-5D-5L-QoL	9.28	7.60	7.20 vs 8.24 0.310 (− 3.08–1.00)
Visual symptoms	8.39	6.82	7.65 vs 6.29 0.142 (− 0.48–3.21)
Endocrine symptoms	34.7	27.9	26.3 vs 29.8 0.346 (− 11.0–3.93)
Nasal symptoms	6.44	5.57	5.85 vs 5.48 0.660 (− 1.33–2.08)

*EQ-5D-5L-QoL* EuroQol-5 dimention-5 level-quality of life, *POS* Pituitary outcome score

## Discussion

### Principal findings

We present the first validated PROM for patients with pituitary adenoma undergoing transsphenoidal surgery. The POS comprises 25 items and is divided into four subscales reflecting core domains in this patient group: EQ-5D-5L-QoL, Visual Symptoms, Endocrine Symptoms, and Nasal Symptoms. The questionnaire’s reliability, responsiveness, validity, and interpretability were assessed systematically using the COSMIN checklist [24] and validated against the SF-36, the most used QoL measure for patients with pituitary adenomas [12]. PROMs are widely recognised as transformational healthcare tools, harnessing the patient’s perspective of symptoms, QoL and functional status to measure the effects of healthcare delivery [16]. Their applications are multitudinous, particularly with the prospect of providing standardised outcome measures that are closely aligned with patient objectives [16]. The POS offers a novel tool to report specific and relevant outcomes measures for this patient group, which will form a valuable adjunct to the established outcome assessments of pituitary patients [6–9].

### Reliability

All subscales showed acceptable internal consistency aside from the Nasal subscale, which may be attributed to the low number of items within the subscale [41, 42]. The POS demonstrated excellent test–retest reliability, with all ICC values above 0.90. Our findings are in keeping with similar validation studies in other fields [43, 44] and demonstrate POS’s reliability.

### Validity

Construct validity was assessed through correlations between the POS subscales and the SF-36 [45]. The POS EQ-5D-5L-QoL subscale had the strongest correlation with SF-36 overall, demonstrating convergent validity with a commonly used and validated QoL measure. The remaining subscales assess domains specific to pituitary adenoma patients undergoing transsphenoidal surgery, exemplified by the low correlations seen between the Visual Symptoms and Nasal Symptoms subscales and SF-36. The Endocrine Symptoms subscale showed moderate to strong correlations with the SF-36 subscales, all below − 0.50, likely due to the POS subscale measuring clinical effects of pituitary conditions strongly affecting QoL [10, 14]. The strongest correlations for this subscale were found with the SF-36 Social Functioning, Vitality, and Mental Health subscales, likely relating to the items related to fatigue, sleep, memory, concentration, sleep, endocrine-related mood swings in the POS Endocrine Symptoms subscale. The lowest correlation seen with the Endocrine Symptoms and SF-36 was due to the SF-36 Physical Functioning subscale, explained by the fact that there are no items directly related to physical activity and function in the POS Endocrine Symptoms. The associations and discrepancies give credence to the POS as a valid PROM, considering the SF-36 is the most employed PROM in pituitary patients [12].

### Responsiveness

To assess changes in effect over time, the sample was divided based on the GPE scale responses for the 6-month (n = 38) post-surgery surveys. Overall patients who self-reported as “better” or “much better” showed greatest improvement in the POS subscales, suggesting that the POS is responsive to detecting clinical changes after surgery. Interestingly, even in the “deteriorated” group there were improvements in the POS subscales compared to pre-operatively, suggesting improvements in symptoms. This discrepancy may be related to patient expectations and should be investigated in future research. We caveat these findings recognising the limitation posed by the small sample in the “no change” and “deteriorated” groups, making it difficult to determine the true responsiveness of the POS questionnaire. Considering GPE responses were skewed towards more improved health states on the last survey, there is a strong inference that most participants did experience improved health states postoperatively and allude towards the POS’s potential in measuring the long-term impacts of surgery.

### Interpretability

To calculate the MIC and MID, values from the responsiveness analysis were used, leading to similar limitations for these calculations. It is not possible to determine whether the observed changes were due to clinically important changes or measurement errors, despite the analyses showing the MIC was smaller than the SDC (ind). However, the SEM and SDC values by themselves could help interpret the POS subscale scores. The pre-operation survey showed no floor and ceiling effect, except for the Nasal Symptoms subscale which had a floor effect, indicating the POS has an appropriate response range. The floor effect for the Nasal subscale at baseline is appropriate patients would not be expected to experience nasal symptoms prior to surgery as this is a possible side effect specific to transsphenoidal surgery. The 6-month post-operation survey did not have a ceiling effect for any of the POS subscales but showed a floor effect for the EQ-5D-5L-QoL, Visual Symptoms and Nasal Symptoms subscales. This is unsurprising for the two former subscales as patients experience improvements in QoL, symptoms and overall health after surgery and thus report minimal symptoms. The floor effect for the Nasal Symptoms subscale on the 6-month post-operation survey is in keeping with previous assertions that nasal symptoms tend to be transient and recover [46, 47]. Overall, these results point towards a good interpretability of the item scales.

### Other analyses

There were no differences in any subscale score between patients who underwent endoscopic surgery and patients who underwent microscopic surgery. However, the sample size for this was small as clinical data and the 6-month post-op survey results (endoscopic surgery n = 20, microscopic surgery n = 21) had to be available for the analyses. This in turn limits the reliability of these results.

Further analysis on patient outcomes measured with the POS is a priority for future research with larger sample sizes. As the aim of the current study was to develop and validate the POS as a tool, the study was not powered for assessing patient outcomes.

### Comparison to related studies

The POS represents the first PROM for patients with pituitary adenoma undergoing transsphenoidal surgery. However, other PROMS have been widely used within this patient group, notably the LBNQ-Pituitary [21] and Anterior Skull Base Questionnaire (ASBQ) for patients with pituitary disease [48] and patients undergoing anterior skull base surgery, respectively [49].

The LBNQ-Pituitary [21] assesses how much patients are affected by their pituitary disease, with five subscales including mood problems, negative illness perceptions, issues in sexual functioning, physical and cognitive complaints, and issues in social functioning. The scale can also be extended with additional optional items, including one on impaired eyesight, and others on specific conditions. However, the LBNQ-Pituitary lacks items related nasal symptoms resulting from the transsphenoidal approach.

The ASBQ assesses how much patients are affected by their anterior skull base surgery, with six subscales including role of performance, physical functioning, vitality, pain, specific symptoms, and impact on emotions [49]. It includes items on nasal symptoms, and one on the effect of surgery on vision, but lacks items dedicated to endocrine symptoms associated with pituitary disease.

The POS differs from both the LBNQ-Pituitary and ASBQ in providing items related to both endocrine and visual symptoms resulting from pituitary disease, and nasal symptoms resulting from transsphenoidal surgery.

### Limitations

This is the first study to validate a PROM for patients with pituitary adenoma undergoing transsphenoidal surgery, using an established standard to inform its design. There are limitations:

1. Six patients did not undergo surgery during the study period due to effects of the pandemic [50]. Three patients had alternate diagnoses.
2. The pandemic resulted in a smaller than planned sample size. Many patients required treatment via emergency rather than elective pathways, impacting particularly on the responsiveness and interpretability analyses. According to the COSMIN checklist, a minimum sample size of 100 is needed for assessing certain properties to obtain valid and reliable results, meaning the true effect sizes of the POS are likely underestimated.
3. Whilst a minimum sample size of 50 is cited for EFA [51], several studies suggest larger sample sizes are required [52–54]. Despite the sample size (n = 92), EFA was performed but the results were used as a guidance and acted on in the context of clinical and rational judgement.

### Recommendations for use

The POS offers a standardised, patient-centric outcome measure for patients having transsphenoidal surgery for pituitary tumours.

We recommend interpreting the subscale scores on an individual basis as the items within each subscale are designed to measure constructs specific to pituitary adenoma patients undergoing transsphenoidal surgery. As the number of items is not evenly distributed across the four subscales, quantification of individual item weights is needed before using the total POS score. The EQ-5D-5L-QoL, Visual Symptoms, and Endocrine Symptoms subscales measure constructs directly related to the pituitary adenoma and consequently the subscale scores are expected to improve after surgery. Conversely, the Nasal Symptoms subscale measures a post-surgical effect from the transsphenoidal surgery itself rather than the condition.

Due to the limited sample size for the responsiveness and interpretability analyses, the POS subscale scores should be interpreted using clinical judgement, particularly for clinically significant change scores, until the literature can suggest more reliable values.

Users should be aware of the differences in total scores for each POS subscale and comparison between POS subscale scores should be done with caution.

## Conclusion

The POS is the first PROM specific for patients undergoing transsphenoidal surgery for a pituitary adenoma. This 25-item PROM with EQ-5D-5L-QoL, Visual Symptoms, Endocrine Symptoms, and Nasal Symptoms subscales demonstrated good measurement properties, offering a new measure to examine surgical effectiveness and sequelae of surgery in a structured fashion. The POS can be integrated into contemporary practice to provide patient-centred assessments of care delivery and treatment efficacy. It can also support research aiming to understand the impacts of transsphenoidal surgery, and supplement quality improvement strategies to transform care delivery by providing standardised outcome measures which are more closely aligned with patient objectives.

## Supplementary Information

Below is the link to the electronic supplementary material.

Supplementary file1 (PDF 117 KB)

Supplementary file2 (DOCX 19 KB)

## Data Availability

The data generated and analysed during the current study are not publicly available due to participant confidentiality but are available upon reasonable request and for three years from the date of publication.

## Declarations

### Copyright & trademarking

© EuroQol Research Foundation. EQ-5D™ is a trademark of the EuroQol Research Foundation. SF-5D was reproduced by permission of EuroQol Research Foundation. Reproduction of this version is not allowed. For reproduction, use or modification of the EQ-5D (any version), please register your study by using the online EQ registration page: www.euroqol.org.
