# Supplementary material for: A patient-reported outcome measure for patients with pituitary adenoma undergoing transsphenoidal surgery
Source: Pituitary. 2022 Jul 15;25(4):673–83. doi: 10.1007/s11102-022-01251-x (PMC9345822; doi:10.1007/s11102-022-01251-x)
Supplement: Supplementary file 1 — Supplementary file1 (PDF 117 KB) [file 11102_2022_1251_MOESM1_ESM.pdf]

A Patient-Reported Outcome Measure for Patients with Pituitary Adenoma Undergoing Transsphenoidal Surgery

Pituitary

Elika Karvandi BSc[1,2], John Gerrard Hanrahan MBBS[2,3], Danyal Z Khan[2,3], Pierre-Marc Boloux[4], Fion Bremner[5], Ivan Cabrilo[2], Neil Dorward[2], Joan Grieve[2], Sue Jackson PhD [6], Glenda Jimenez[2], Inma Serrano[2], Victoria Anne Nowak[5], Angelos Kolias[1,2], Stephanie E Baldeweg[4,7*], Hani Joseph Marcus[2,3*].

Affiliations

1. Department of Neurosurgery, University of Cambridge, Cambridge, UK.
2. Division of Neurosurgery, National Hospital for Neurology and Neurosurgery, London, UK.
3. Wellcome / EPSRC Centre for Interventional and Surgical Sciences, University College London, London, UK.
4. Department of Diabetes & Endocrinology, University College London Hospitals NHS Foundation Trust, London, UK.
5. Department of Neuro-ophthalmology, National Hospital for Neurology and Neurosurgery, London, UK.
6. Department of Psychology, University of Plymouth, Plymouth, UK.
7. Centre for Obesity and Metabolism, Department of Experimental and Translational Medicine, Division of Medicine, University College London, London, UK.

*= joint senior authors

Author Correspondence: Elika Karvandi, Department of Neurosurgery, University of Cambridge, Cambridge, UK. eek34@cam.ac.uk

Supplementary Material

Supplementary Material 1: Global Perceived Effect (GPE) scales

Survey 1: In general, how much would you say your pituitary tumour affects your health?

Survey 2: In general, how would you say your pituitary symptoms are compared to when you last completed the survey (approximately two weeks ago)?

Survey 3: In general, how would you say your pituitary symptoms are compared to before the surgery (approximately 3 months ago)?

Survey 4: In general, how would you say your pituitary symptoms are compared to before the surgery (approximately 6 months ago)?
