# Supplementary material for: A patient-reported outcome measure for patients with pituitary adenoma undergoing transsphenoidal surgery
Source: Pituitary. 2022 Jul 15;25(4):673–83. doi: 10.1007/s11102-022-01251-x (PMC9345822; doi:10.1007/s11102-022-01251-x)
Supplement: Supplementary file 2 — Supplementary file2 (DOCX 19 KB) [file 11102_2022_1251_MOESM2_ESM.docx]

## **Panel: The Pituitary Outcome Score**

**EQ-5D-5L-QoL (lowest score = 5, highest score = 25)**

Under each heading, please tick the one box that best describes your health today.

**Mobility**

1. I have no problems in walking about
2. I have slight problems in walking about
3. I have moderate problems in walking about
4. I have severe problems in walking about
5. I am unable to walk about

**Self-care**

1. I have no problems washing or dressing myself
2. I have slight problems washing or dressing myself
3. I have moderate problems washing or dressing myself
4. I have severe problems washing or dressing myself
5. I am unable to wash or dress myself

**Usual activities (e.g. work, study, housework, family or leisure activities)**

1. I have no problems doing my usual activities
2. I have slight problems doing my usual activities
3. I have moderate problems doing my usual activities
4. I have severe problems doing my usual activities
5. I am unable to do my usual activities

**Pain or discomfort**

1. I have no pain or discomfort
2. I have slight pain or discomfort
3. I have moderate pain or discomfort
4. I have severe pain or discomfort
5. I have extreme pain or discomfort

**Anxiety or depression**

1. I am not anxious or depressed
2. I am slightly anxious or depressed
3. I am moderately anxious or depressed
4. I am severely anxious or depressed
5. I am extremely anxious or depressed

**Visual Symptoms (lowest score = 4, highest score = 21)**

Please think about your eyesight in the past month. If you use glasses or contact lenses for some activities, please answer according to how you can see when using them.

**How often have you experienced double vision?**

1. Never
2. Some of the time
3. Most of the time
4. All of the time

**How much has your eyesight interfered with your life in general?**

1. Not at all
2. Hardly at all
3. A little
4. A fair amount
5. A lot
6. An extremely large amount

**How would you describe your vision – with both eyes open, wearing glasses or contact lenses if you usually do?**

1. Excellent
2. Very good
3. Quite good
4. Average
5. Quite poor
6. Very poor
7. Appalling

**How often has your eyesight prevented you from doing things you would like to do?**

1. Never
2. Some of the time
3. Most of the time
4. All of the time

**Endocrine Symptoms (lowest score = 13, highest score = 78)**

Please think about your health in the past month. N.B. If any of the items are not applicable to you, please select ***“no problem at all”***

**How much are you affected by fatigue?**

1. No problem at all
2. Very mild problem
3. Mild or slight problem
4. Moderate problem
5. Severe problem
6. Problem as bad as it can be

**How much are you affected by light-headedness?**

1. No problem at all
2. Very mild problem
3. Mild or slight problem
4. Moderate problem
5. Severe problem
6. Problem as bad as it can be

**How much are you affected by loss of libido or sexual function?**

1. No problem at all
2. Very mild problem
3. Mild or slight problem
4. Moderate problem
5. Severe problem
6. Problem as bad as it can be

**How much are you affected by fertility problems?**

1. No problem at all
2. Very mild problem
3. Mild or slight problem
4. Moderate problem
5. Severe problem
6. Problem as bad as it can be

**How much are you affected by changes in your appetite?**

1. No problem at all
2. Very mild problem
3. Mild or slight problem
4. Moderate problem
5. Severe problem
6. Problem as bad as it can be

**How much are you affected by changes in your weight?**

1. No problem at all
2. Very mild problem
3. Mild or slight problem
4. Moderate problem
5. Severe problem
6. Problem as bad as it can be

**How much are you affected by increased thirst and frequent urination?**

1. No problem at all
2. Very mild problem
3. Mild or slight problem
4. Moderate problem
5. Severe problem
6. Problem as bad as it can be

**How much are you affected by dry, thin, or easily bruised skin?**

1. No problem at all
2. Very mild problem
3. Mild or slight problem
4. Moderate problem
5. Severe problem
6. Problem as bad as it can be

**How much are you affected feeling too hot or cold?**

1. No problem at all
2. Very mild problem
3. Mild or slight problem
4. Moderate problem
5. Severe problem
6. Problem as bad as it can be

**How much are you affected by memory problems?**

1. No problem at all
2. Very mild problem
3. Mild or slight problem
4. Moderate problem
5. Severe problem
6. Problem as bad as it can be

**How much are you affected by problems concentrating?**

1. No problem at all
2. Very mild problem
3. Mild or slight problem
4. Moderate problem
5. Severe problem
6. Problem as bad as it can be

**How much are you affected by problems sleeping?**

1. No problem at all
2. Very mild problem
3. Mild or slight problem
4. Moderate problem
5. Severe problem
6. Problem as bad as it can be

**How much are you experiencing mood swings?**

1. No problem at all
2. Very mild problem
3. Mild or slight problem
4. Moderate problem
5. Severe problem
6. Problem as bad as it can be

**Nasal Symptoms (lowest score = 1, highest score = 18)**

Please think about your health in the past month. N.B. If any of the items are not applicable to you, please select ***“no problem at all”***

**How much are you affected by nasal congestion?**

1. No problem at all
2. Very mild problem
3. Mild or slight problem
4. Moderate problem
5. Severe problem
6. Problem as bad as it can be

**How much are you affected by an altered sense of smell or taste?**

1. No problem at all
2. Very mild problem
3. Mild or slight problem
4. Moderate problem
5. Severe problem
6. Problem as bad as it can be

**How much are you affected by headaches?**

1. No problem at all
2. Very mild problem
3. Mild or slight problem
4. Moderate problem
5. Severe problem
6. Problem as bad as it can be
